# The effect of chronic prenatal hypoxia on the development of mature neurons in the cerebellum

**DOI:** 10.1186/1866-1955-5-17

**Published:** 2013-07-03

**Authors:** Keumyoung So, Yoonyoung Chung, Hyunyoung Lee, Eunyoung Kim, Yonghyun Jeon

**Affiliations:** 1 Department of Anesthesiology and Pain medicine, Chosun University Hospital, 375 Seosuk-dong, Dong-Gu, Gwangju 501-759, Korea; 2 Department of Anatomy, School of Medicine, Chosun University, 375 Seosuk-dong, Dong-Gu, Gwangju 501-759, Korea; 3 Department of pediatrics, Chosun University Hospital, 375 Seosuk-dong, Dong-Gu, Gwangju 501-759, Korea

**Keywords:** Hypoxia, Cerebellum, Guinea pig

## Abstract

**Background:**

Adverse intrauterine circumstances can result in abnormal brain development, and can contribute to many neurological disorders such as cerebral palsy and cognitive and behavioral deficits. These neurological problems are caused by conditions that cause chronic placental insufficiency (CPI), such as hypoxia and acidemia. Hypoxia has been implicated in structural alterations of the cerebellum during development; however, the changes to the cerebellar external granular layer (EGL) induced by chronic prenatal hypoxia are not well understood. We therefore investigated the effect of chronic prenatal hypoxia on the development of mature neurons in the EGL using the guinea pig CPI model.

**Methods:**

Unilateral uterine artery ligation was performed at 30 to 32 days of gestation (dg) - with term defined as approximately 67 dg. At 50 dg, 60 dg, and one week after birth, fetuses and newborns were sacrificed and assigned to either the growth-restricted (GR) or control (no ligation) group. After fixation, dissection, and sectioning of cerebellar tissue from these animals, immunohistochemistry was performed with antibodies raised to hypoxia-induced factor 1α (Hif1α), Pax6, NeuroD, and NeuN.

**Results:**

The induction of hypoxia was confirmed by the presence of Hif1α immunoreactivity in the EGL of the GR (but not control) fetuses. The only other cellular immunoreactivity found in any of the tissues was to the NeuN antibody, which is a marker of mature neurons. The proportion of NeuN-immunoreactive (NeuN-IR) cells to the total number of cells in the EGL did not differ between the GR and control groups at 50 and 60 dg. The density of NeuN-IR cells was greater in GR fetuses than in controls at 60 dg (*P* < 0.05) but not at 50 dg. At one week after birth, the EGL was just one cell thick, and only a few NeuN-IR cells could be observed in both groups. TUNEL assays performed to enable the evaluation of apoptosis in the cerebellar EGL revealed that cell death was not affected by hypoxia at 50 dg, 60 dg, and one week after birth.

**Conclusion:**

These findings indicate that chronic prenatal hypoxia affects the process of neuronal production late in fetal life, but that this effect does not persist postnatally.

## Background

An inadequate intrauterine environment is thought to affect fetal brain development [1,2]. Very low birth weight, which is affected by intrauterine growth restriction (IUGR), is associated with a regional reduction in brain volume [3] and an increased risk of white matter injury [4].

Abnormal brain development is associated with neurological disorders such as cerebral palsy [5], schizophrenia [6], and cognitive [7] and learning/memory [8] deficits. These neurological problems are affected by conditions that cause chronic placental insufficiency (CPI), such as malnutrition, hypoxia, and acidemia [9]. Several models of CPI have been used to determine the relationship between neurological disorders and abnormal brain development. For example, Duncan *et al*. reported that late gestational CPI affects the morphology and neurotrophin expression of the postnatal sheep brain [10]. Moreover, IUGR induced by CPI in the guinea pig was shown to affect the maturation of myelin [11].

In a recent guinea pig model of CPI, which was induced by uterine artery ligation, the growth of the cellular and neuropil layers were found to be reduced in the cerebellum, without concomitant changes in the expressions of immunoreactivity to either brain-derived neurotrophic factor (BDNF)-IR or tropomyosin receptor kinase B (TrkB)-IR [12]. Some studies have implicated hypoxia in structural alterations of the cerebellum during development. First, significant reductions have been observed in the width of the molecular and inner granular layers [13]. Second, the size and number of Purkinje cells were also found to be reduced [14]. Unlike changes in other layers of the cerebellum, the changes in the cerebellar external granular layer (EGL) induced by chronic prenatal hypoxia are not well understood. Granule cells comprise half of the neurons in the central nervous system, and their precursors are dispersed within the cerebellar EGL [15].

The effects of hypoxia on the development of mature neurons in the EGL were investigated in the present study using a guinea pig CPI model.

## Methods

### Animal surgery

All of the animal experiments were approved by Chosun University Institutional Animal Care and Use Committee (approval number CIACUC2012-A0007). Pregnant Dunkin-Hartley guinea pigs were obtained from a certified breeder (Central Laboratory Animals, Korea). Unilateral uterine artery ligation was performed as reported by Mallard *et al*. [16]. Briefly, animals were anesthetized with Zoletil (10 mg/kg; Virbac, France) and xylazine (0.15 mg/kg; Bayer, Germany), which were administered via intramuscular injection at 30 to 32 days of gestation (dg) - with term defined as approximately 67 dg. After shaving, a midline incision was made below the umbilicus under aseptic conditions. The fat pad of one of the uterine horns was exposed and ligated with silk sutures (4/0). After the procedure, the abdomen was disinfected with a solution of povidone-iodine (Green Medical, Korea). The animals (*n* = 28) were then caged and raised in the same environment.

### Tissue preparation

Fetuses were delivered by cesarean section at 50 dg (*n* = 14) and 60 dg (*n* = 14). Fetuses from the unoperated horn were assigned to the control group (50 dg, *n* = 7; 60 dg, *n* = 7), and those from the other, ligated horn were assigned to the growth-restricted (GR) group (50 dg, *n* = 7; 60 dg, *n* = 7). On removal from the uterine horn, fetuses were fixed in 4% paraformaldehyde (PFA) solution. Fetal cerebella were separated from their brainstem and postfixed in 4% PFA at 4°C. After two days, the cerebella were washed with water, dehydrated through a graded ethanol series, and then embedded in paraffin. Serial 12 μm-thick sections of the cerebellum were mounted on gelatin-coated slides (Fisher Scientific, USA).

### Immunohistochemistry

The sections were deparaffinized, rehydrated, and then washed in 0.1 M PBS (pH 7.4). A microwave antigen-retrieval step was performed with 0.01 M sodium citrate buffer (pH 6.0). After cooling, the sections were treated with 0.3% hydrogen peroxide for 20 minutes to block endogenous peroxidase and then rinsed in PBS. They were blocked with normal horse serum in 0.5% BSA solution for 30 minutes at room temperature and then incubated with one of the following primary antibodies overnight at 4°C: rabbit anti-hypoxia-induced factor 1α (Hif1α; 1:500, Abcam, UK), rabbit anti-Pax6 (1:1,000, Abcam), goat anti-NeuroD (1:100, Santa Cruz, USA), and mouse anti-NeuN (1:100; Millipore, USA). The next day, the sections were rinsed several times in PBS, and the immunoreactivity visualized using a biotinylated anti-mouse IgG, the avidin-biotin-peroxidase (ABC) detection system (Vectastain ABC Elite Kit, Vector Laboratories), and the chromogen 3,3’-diamino-benzidine. Thionin counterstaining was performed, after which the sections were dehydrated and coverslipped using PolyMount mounting medium (Polysciences, USA).

### TUNEL staining

Terminal deoxynucleotidyl transferase dUTP nick end labeling (TUNEL) assays were performed with the ApopTag Peroxidase *In Situ* Apoptosis Detection Kit (S7100, Millipore) according to the manufacturer’s protocol.

### Quantification

The slides containing stained sections were viewed via a digital CCD camera that was mounted on a light microscope (Olympus BX41, USA). The number of NeuN- and Hif1α-immunoreactive (IR) cells in the EGL of cerebellar lobules I and VIII were counted (immunoreactivity to the other tested antibodies was not observed). The cerebellum was bisected in the midsagittal plane and one-half was embedded in paraffin with the vermal surface as the cutting surface [17]. Sagittal sections of cerebellum that were separated by 300 μm were chosen from each animal as described previously [12]. The densities of NeuN-IR cells in the EGL of lobules I and VIII were determined using a counting frame of a defined size placed over five areas selected randomly in each section, and quantified in cells/mm^2^. The proportion of NeuN-IR cells was expressed as a percentage of the total number of cells in the EGL.

### Statistical analysis

All data were analyzed using the Statistical Package for Social Sciences (Information Analysis Systems, SPSS, USA). All measurements were compared between the control and GR groups using Student’s *t*-test. The level of statistical significance was set at *P* < 0.05.

## Results

### At 50 dg

**Figure 1:**
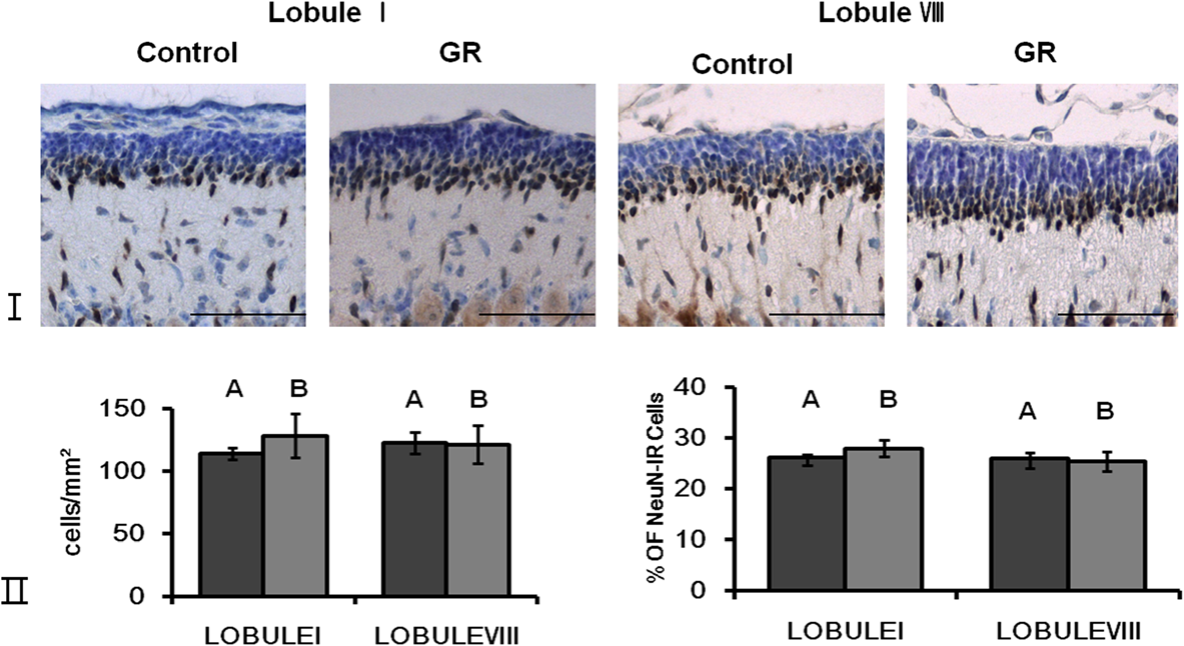
**Representative photomicrographs of NeuN-IR cells in the EGL at 50 dg. (I)** NeuN-IR cells were located at the bottom of the EGL. Scale bars= 100 μm. **(II)** The density and proportion of NeuN-IR cells in the EGL of lobule I and lobule VIII from controls and GR fetuses at 50 dg (A: Control B: GR). The density and proportion of NeuN-IR cells in the EGL did not differ significantly between control and GR fetuses. Values are expressed as a Mean ± SEM. (p > 0.05).

**Figure 2:**
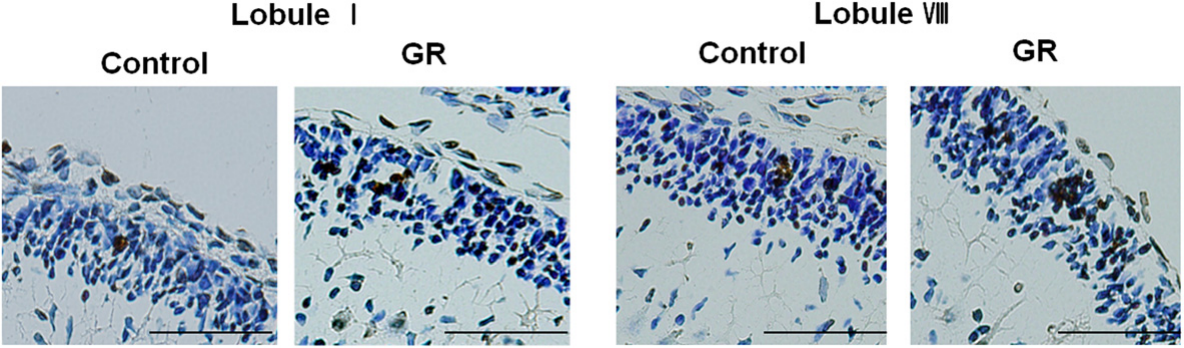
**Representative photomicrographs of TUNEL-positive cells in the external granular layer (EGL) at 50 dg.** TUNEL-positive cells were seen only rarely in the EGL of both groups. Scale bars = 100 μm.

**Figure 3:**
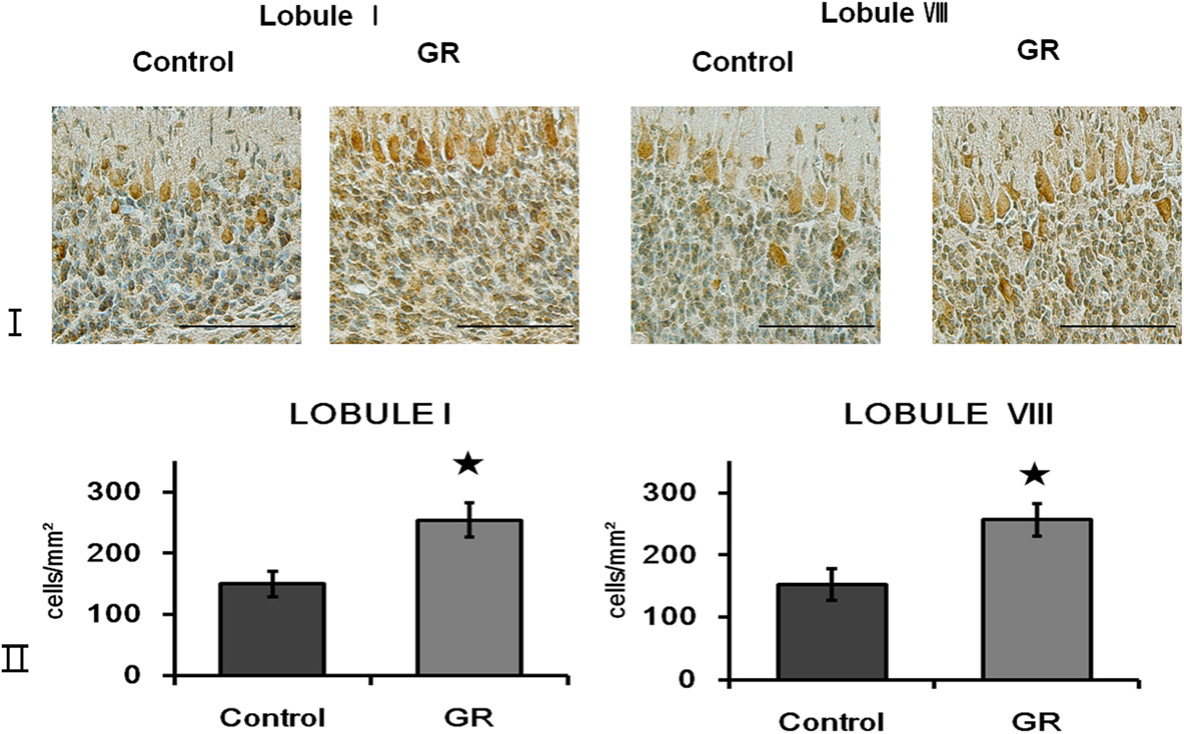
**Expression of Hif1α in the IGL at 50 dg. (I)** Hif1α Immunoreactivity in the IGL of lobule I and VIII from a control and a GR fetus at 50 dg. Scale bars= 100 μm. **(II)** The density of Hif1α-IR cells in the IGL of lobule I and lobule VIII from controls and GR fetuses at 50 dg. The density of Hif1α-IR cells was greater in the GR fetuses than in the controls. Values are expressed as a Mean ± SEM. ★ p<0.05.

There were no Pax6-IR or NeuroD-IR cells in the EGL at 50 dg (data not shown). NeuN-IR cells were located at the bottom of the EGL (Figure 1). The density and relative proportion (Figure 1) of NeuN-IR cells in the EGL did not differ significantly between control and GR fetuses. TUNEL-positive cells were seen only rarely in the EGL of both groups (Figure 2). The density of Hif1α-IR cells was greater in the GR fetuses than in the controls (*P* < 0.05; Figure 3).

### At 60 dg

**Figure 4:**
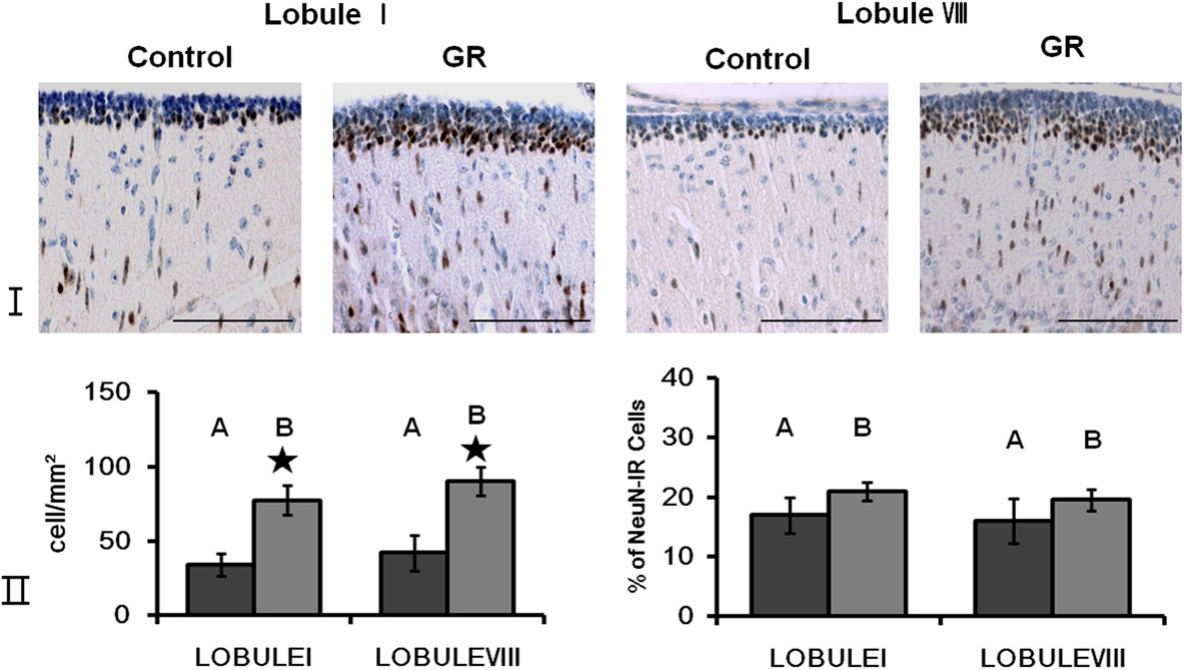
**The number of mature neurons in the EGL was greater in GR fetuses than in the controls at 60 dg. (I)** Representative photomicrographs of the NeuN immunoreactivity in the EGL of lobule I and VIII from a control and a GR fetus at 60 dg. Scale bars= 100 μm. **(II)** The density of NeuN-IR cells in the EGL of lobule I and lobule VIII from controls and GR fetuses at 60 dg (A: Control B: GR). The density of NeuN-IR cells was increased in both lobules of GR fetuses compared to controls at 60 dg. However the proportion of NeuN-IR cells did not differ in both lobules of GR fetuses compared to controls at 60 dg. Values are expressed as a Mean ± SEM. ★ p< 0.05.

**Figure 5:**
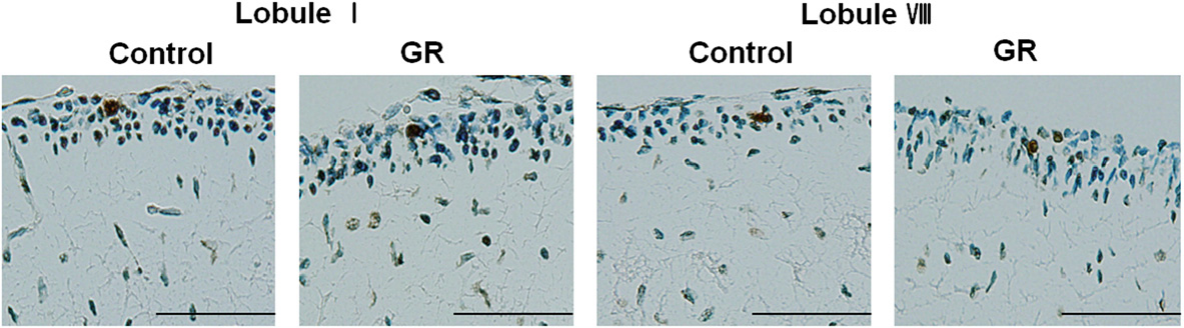
**TUNEL-positive cells in the external granular layer (EGL) of lobule I and VIII from a control and a growth-restricted (GR) fetus at 60 dg.** Few TUNEL-positive cells could be observed in the EGL of either group. Scale bars = 100 μm.

**Figure 6:**
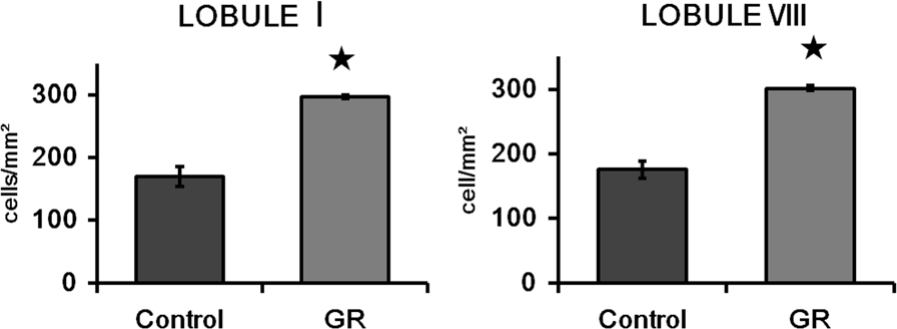
**The density of Hif1α-immunoreactive (Hif1α-IR) cells in the internal granular layer (IGL) of lobule I and lobule VIII from controls and growth-restricted (GR) fetuses at 60 dg.** The density of Hif1α-IR cells was greater in the GR fetuses than in the controls fetus. Values are expressed as mean ± SEM. Black star *P* < 0.05.

There were also no Pax6-IR or NeuroD-IR cells in the EGL at 60 dg. Furthermore, the pattern of cerebellar NeuN immunostaining in 60-dg fetuses was similar to that in 50-dg fetuses (Figure 4). The density of NeuN-IR cells was greater in GR fetuses than in controls at 60 dg (*P* < 0.05; Figure 4). The relative proportion of NeuN-IR cells in the EGL did not differ significantly between the two groups (Figure 4). Few TUNEL-positive cells could be observed in the EGL of either group (Figure 5). The density of Hif1α-IR cells was greater in GR fetuses than in controls (*P* < 0.05; Figure 6), and did not differ significantly from that in 50-dg fetuses.

### At one week after birth

At one week after birth, the EGL was forming a one cell-thick layer in both of the lobules examined. NeuN-IR cells were only rarely observed in the EGL (data not shown), and Pax6-IR and NeuroD-IR cells could not be found at all. As with NeuN-IR, TUNEL-positive cells were observed only very rarely in the EGL (data not shown).

## Discussion

The guinea pig is a useful model of brain development because it has a relatively long gestation period (with term occurring at approximately 67 dg) and most of the development occurs *in utero*. Lobules I and VIII were selected for examination because they are the first and one of the last lobules respectively, to mature [18]. Unilateral uterine artery ligation was performed at 30 dg, and we studied fetuses at 50 and 60 dg. It has been reported previously that the cerebellum of 30-dg fetuses appeared immature and consisted of undifferentiated neurons [16]. Furthermore, Jansson *et al*. [19] reported that fetal weight and placental blood flow were decreased in guinea pigs at 45, 55, and 65 dg. Therefore, we expected that neurogenesis in the guinea pig cerebellum would be reduced at 50 and 60 dg. However, the relative proportions of NeuN-IR cells did not differ significantly between control and GR animals at 50 and 60 dg, and the pattern of immunoreactivity was similar between 50 and 60 dg. On the other hand, the density of NeuN-IR cells was increased in GR fetuses at 60 dg.

NeuN is a neuronal-specific nuclear protein [20] and NeuN immunoreactivity is used to obtain independent estimates of total numbers of neuronal and nonneuronal cells [21]. At 50 dg, the proportion of NeuN-IR cells relative to the total number of cells and the density of NeuN-IR cells in the EGL did not differ between normal and hypoxic cerebella. This suggests that chronic hypoxia does not have a comprehensive effect on neuronal cell formation at 50 dg. However, a significant reduction in the number of NeuN-positive neuronal nuclei was demonstrated in the developing chick brain [22] and Rees *et al*. [23] reported a reduction in the density of cells undergoing mitosis in the EGL layer of hypoxemic fetal sheep compared to controls. The differences in the findings between the present study and others may be at least partly attributable to the use of different species of experimental model and to the degree of hypoxia imposed. The expression of Hif1α is induced by hypoxia-ischemia [24]. Therefore, while the presence of HIF1α immunoreactivity in the fetal tissue at 50 dg demonstrates that at that time the fetus had been in a hypoxic condition, it does not indicate whether that hypoxia was mild or severe. Jansson *et al*. performed unilateral uterine artery ligation at 30 to 32 dg in the guinea pig [19]. Placenta blood flow was reduced by 25% at 45 dg and by almost 50% at 55 dg in the ligated horn [19]. Further experiments are needed to determine the degree of hypoxia that is established using this ligation model, and the degree to which it varies between animals at the same gestation time points. However, since the major developmental events in the guinea pig brain occur before 48 dg [25], it is possible that ligation of the uterine artery affects brain development at 50 dg.

At 60 dg, the proportion of NeuN-IR cells did not differ between control and GR cerebella. However, the density of NeuN-IR cells was increased in GR fetuses, indicating a larger total cell volume. The density of HIF1α-IR cells did not differ between 60 and 50 dg, which implies that the hypoxic condition had not changed between these two time periods. The TUNEL assay findings demonstrate that cell death was not affected by the hypoxia induced in this model. Pax6 is a neuronal stem cell marker [26] and NeuroD is implicated in the differentiation of neurons [27]; we observed no cell immunoreactivity to either of these markers, which suggests that neuronal maturation is already complete at this stage (that is, 60 dg). We performed immunohistochemistry with Pax6 and NeuroD at 30 dg, and observed a few Pax6-IR and NeuroD-IR cells. However, the number of sections was insufficient for providing representative data. An earlier-gestation guinea pig model is required to study the early stages of neuron formation. Since in normal brain development the granule cells in the cerebellar EGL migrate medially and the EGL gradually disappears [28], it is possible that the migration of mature neurons was delayed at this time.

At one week after birth, NeuN-IR cells were only rarely observed in the EGL under both the GR and control conditions. As mentioned above, this is the usual process of cerebellum development. In our study, the observed prenatal alterations to the EGL did not persist postnatally. More studies with animals of different ages are required to establish how chronic prenatal hypoxia affects the mature cerebellum.

## Conclusion

The findings of this study suggest that chronic prenatal hypoxia affects the process of neuronal production late in fetal life, but that this effect does not persist postnatally. The approach used in this study is useful for extending our understanding of neurogenesis in the EGL, and the findings may be useful for elucidating the brain injury caused by prenatal hypoxia.

## Abbreviations

CPI: Chronic placental insufficiency; Dg: Days of gestation; EGL: External granular layer; GR: Growth-restricted; IR: Immunoreactive.

## Competing interests

The authors declare that they have no competing interests.

## Authors’ contributions

YYJ designed the study and performed the statistical analyses. KYS, HYL, and EYK participated in the surgical procedures. YHJ carried out the immunohistochemistry assay and analyzed the obtained data. All authors read and approved the final manuscript.
